# 440. Steroid Use and Elevated Rates of Coinfections and Mortality in Mild-COVID-19 Patients

**DOI:** 10.1093/ofid/ofad500.510

**Published:** 2023-11-27

**Authors:** David E Rebellon-Sanchez, Julio Llanos-Torres, Carolina Álvarez-Ortega, Tania M Guzman-Gonzales, Sarita Rodríguez, Fernando Rosso

**Affiliations:** Fundacion Valle del Lili, Cali, Valle del Cauca, Colombia; Fundacion Valle del Lili, Cali, Valle del Cauca, Colombia; Fundación Valle del Lili, cali, Valle del Cauca, Colombia; Fundación Valle del Lili, cali, Valle del Cauca, Colombia; Fundación Valle del Lili, cali, Valle del Cauca, Colombia; Fundación Valle del Lili, cali, Valle del Cauca, Colombia

## Abstract

**Background:**

Systemic corticosteroids are used to treat patients with COVID-19. Since the findings of the RECOVERY trial, steroids have been broadly used in critical patients. However, their use in patients with mild disease was unclear and may even be harmful. In the hard times of the pandemic in the absence of widely available vaccines or antivirals agents, hospital overcrowding, patients had to be referred to home care and steroids could be overused. The aim of this study was to explore the relationship between the use of corticosteroids, secondary infections and overall mortality in adults with COVID-19

**Methods:**

A prospective observational study was performed in a university hospital in Cali, Colombia. Severity was assessed with NEWS score 2. The study included patients at various levels of severity, including patients admitted to the emergency department, inpatients, and those admitted to the intensive care unit

**Results:**

A total of 12,227 patients were treated at our hospital during the first four waves of the pandemic. 54.8% were female, with median age of 45 years (IQR: 31 - 62). 68.5% were classified as mild COVID-19, 8.8% as moderate, and 22.68% as severe. 5.49% of patients were pregnant. Of all patients, 24.30% received corticosteroids upon admission, 11.6% developed secondary infections, and 8.4% died. Mild COVID-19 cases were 1,988 (wave 1), 2,147 (wave 2), 1,628 (wave 3), and 1,608 (wave 4). The proportions of corticosteroid used by waves were 4.3%, 7.18%, 12.5%, and 8.1%, respectively. Corresponding proportions for secondary infections were 2.80%, 5.26%, 6.14%, and 3.8%, while death proportions were 1.7%, 2.5%, 3.2%, and 2.1%. A positive correlation was found between corticosteroid use and secondary infection and mortality rates (Fig. 1). Correlation coefficients were r= 0.9 (p= 0.09) for corticosteroid use and mortality, r= 0.84 (p= 0.15) for corticosteroid use and secondary infection, and r= 0.94 (p= 0.01) for secondary infection and mortality. These correlations were not observed in moderate and severe COVID-19 cases

Relationship between corticosteroid use, secondary infection and mortality in patients with COVID-19

**Figure:**
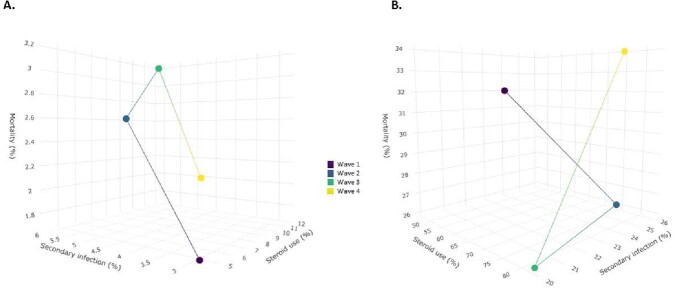

A. Relationship in mild COVID-19 patients. B. Relationship in moderate-to-severe COVID-19 patients

**Conclusion:**

Our study shows that the use of corticosteroids in mild cases could correlate with a greater proportion of secondary infections and mortality

**Disclosures:**

**All Authors**: No reported disclosures

